# Duodenitis associated with ulcerative colitis and pouchitis after total colectomy successfully treated with upadacitinib: A case report

**DOI:** 10.1002/deo2.415

**Published:** 2024-08-05

**Authors:** Kentaro Kojima, Jun Takada, Kiichi Otani, Naoya Masuda, Yukari Tezuka, Sachiyo Onishi, Masaya Kubota, Takashi Ibuka, Masahito Shimizu

**Affiliations:** ^1^ Department of Gastroenterology and Internal Medicine Gifu University Graduate School of Medicine Gifu Japan

**Keywords:** colectomy, duodenitis, pouchitis, ulcerative colitis, upadacitinib

## Abstract

A 27‐year‐old man had ulcerative colitis (UC) 1 year prior and underwent a colectomy and two‐stage ileal pouch‐anal anastomosis for medically refractory UC 6 months ago. He visited our department with epigastric pain and discomfort, increased stool frequency, and bloody diarrhea. Esophagogastroduodenoscopy revealed continuous diffuse friable mucosa, erosions, and edema in the duodenum, and pouchoscopy revealed multiple ulcers and purulent mucus adhesions. Based on endoscopic and pathological findings, the patient was diagnosed with duodenitis associated with UC and pouchitis, for which he received oral prednisolone (40 mg/day) and ciprofloxacin. The frequency of stools and occurrence of bloody diarrhea reduced, and epigastric pain and discomfort improved after 2 weeks. However, when prednisolone was discontinued, the symptoms worsened, albumin level decreased, and C‐reactive protein level increased. Following this, we administered a 20 mg prednisolone sodium phosphate enema once daily, and the patient's symptoms improved. However, the symptoms relapsed when the enema was discontinued. Assuming that the patient had steroid‐dependent duodenitis associated with UC and pouchitis, we initiated upadacitinib. His symptoms improved within a few days, and biomarkers returned to normal after 1 month. Nine months after initiating the upadacitinib treatment, endoscopic remission was achieved in the mucosa of the duodenum and pouch. The patient has been in clinical remission for 1 year without any adverse events.

## INTRODUCTION

Ulcerative colitis (UC) is an idiopathic nonspecific inflammatory disease primarily involving the mucosa and submucosa of the colorectum. UC pathogenesis is unclear but is thought to involve genetic and environmental factors. Some antigens cause an excessive immune response in the intestine and are involved in inflammation.^1^ While Crohn's disease, another inflammatory bowel disease, affects the entire digestive tract, UC affects the colorectum, except in backwash ileitis and pouchitis.

Recently, UC‐related upper gastrointestinal involvements (UGI) have been reported.^2^ This condition is a diffuse inflammation of the stomach and duodenum resembling colitis. An immunological mechanism common to UC is considered to be involved in the onset. A standard treatment for UC‐related UGI has not yet been established as it is rare. However, UC treatments, such as 5‐aminosalicylic acid and corticosteroids, have proven effective.^3^ Additionally, anti‐tumor necrosis factor‐α agents and calcineurin inhibitors are also effective for UC‐related UGI.^4^, ^5^ Here, we report a patient with duodenitis associated with UC and pouchitis after a total colectomy who was successfully treated with upadacitinib (UPA).

### Case report

**FIGURE 1:**
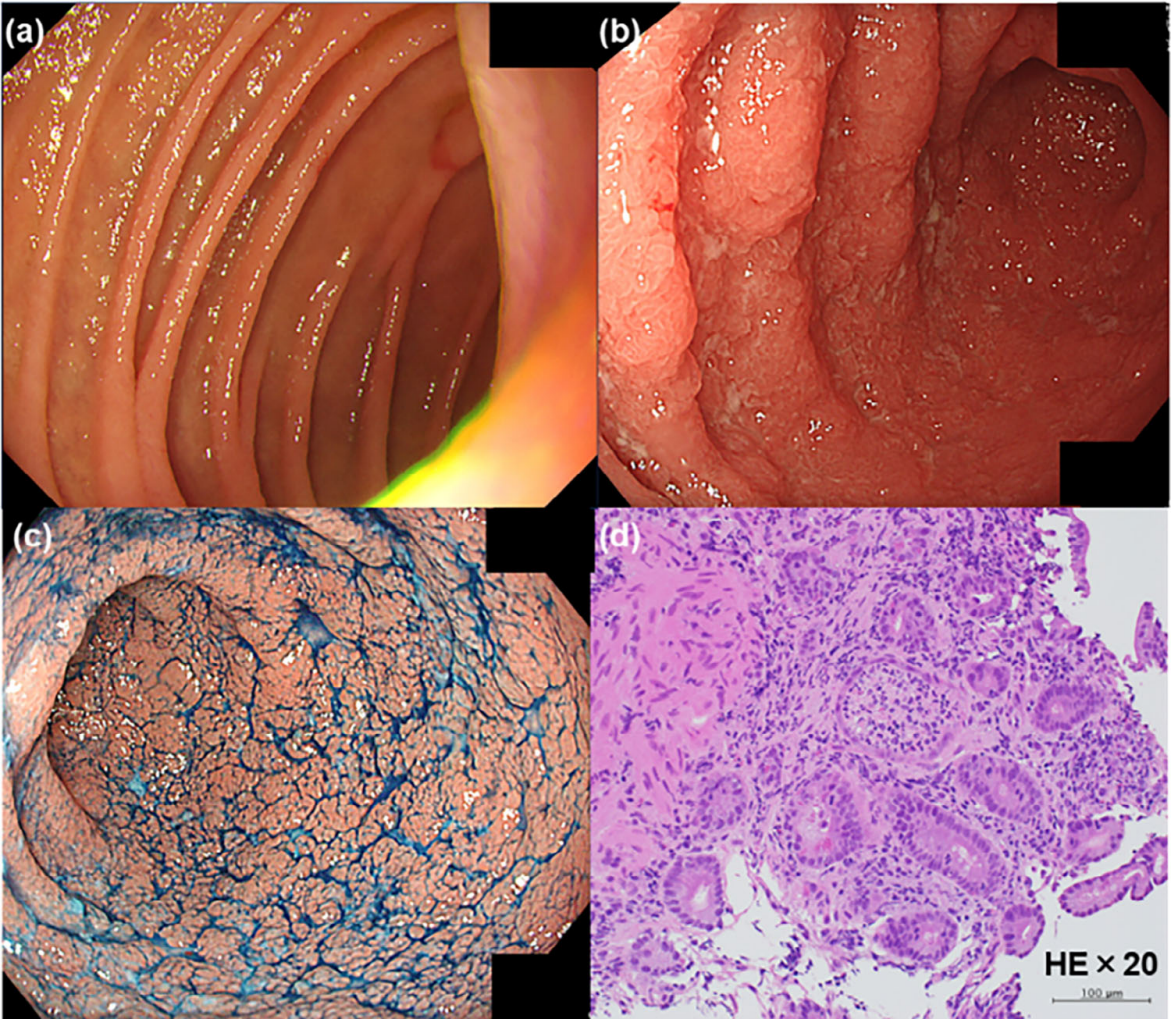
Esophagogastroduodenoscopy revealed no abnormal findings in the duodenum before stoma closure (a) and continuous diffuse friable mucosa and edema (b, c). Biopsy revealed diffuse inflammatory cell infiltration, cryptitis, and crypt abscesses (hematoxylin and eosin staining. Magnification ×20) (d).

**FIGURE 2:**
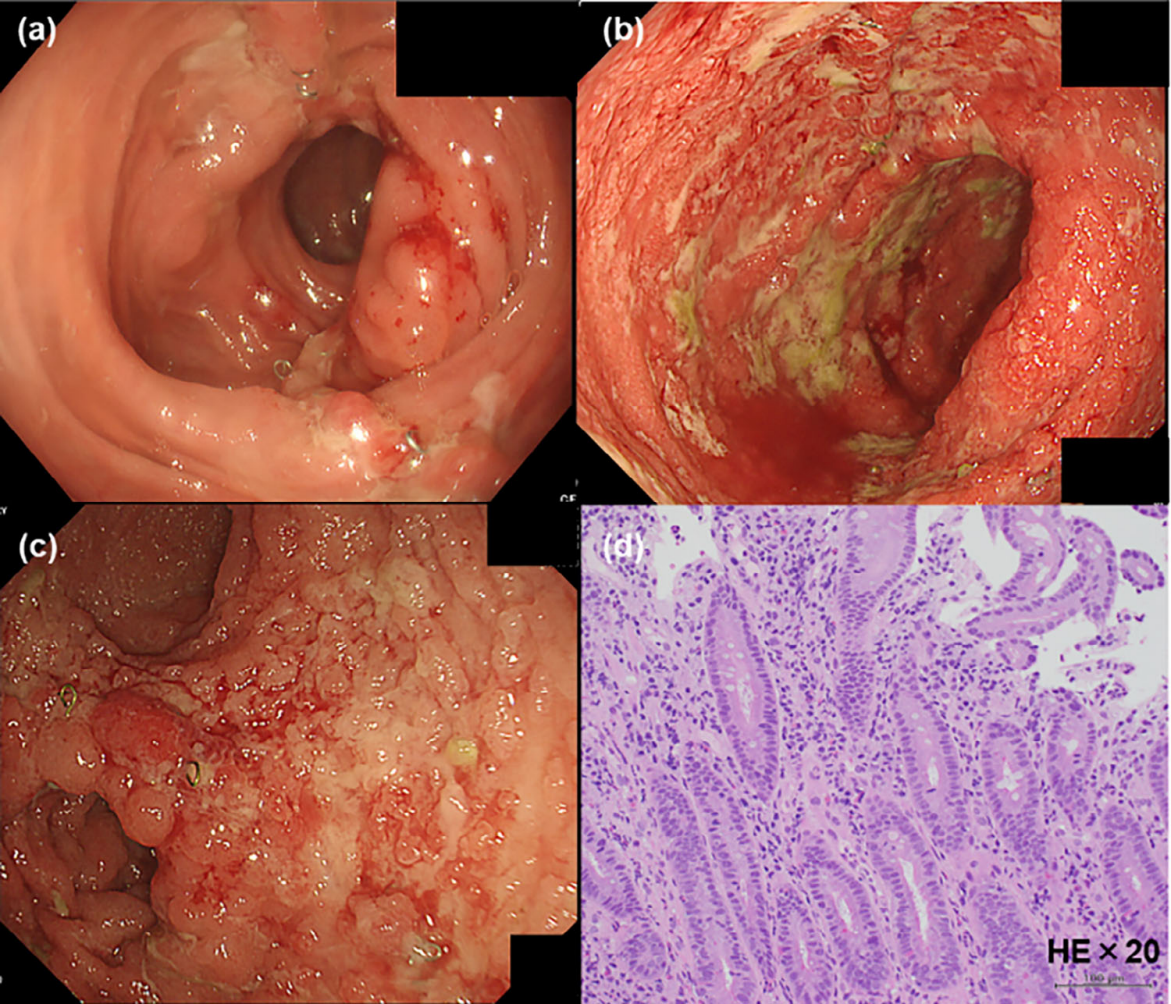
Pouchcoscopy revealed no abnormal findings before stoma closure (a) and multiple ulcers and purulent mucus adhesion when he visited our department (b, c). The biopsy revealed numerous neutrophilic infiltrations (hematoxylin and eosin staining. Magnification ×20) (d).

**FIGURE 3:**
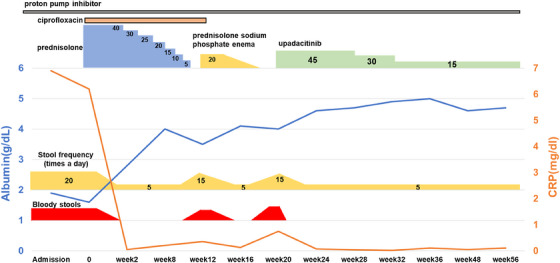
Clinical course of the patient

**FIGURE 4:**
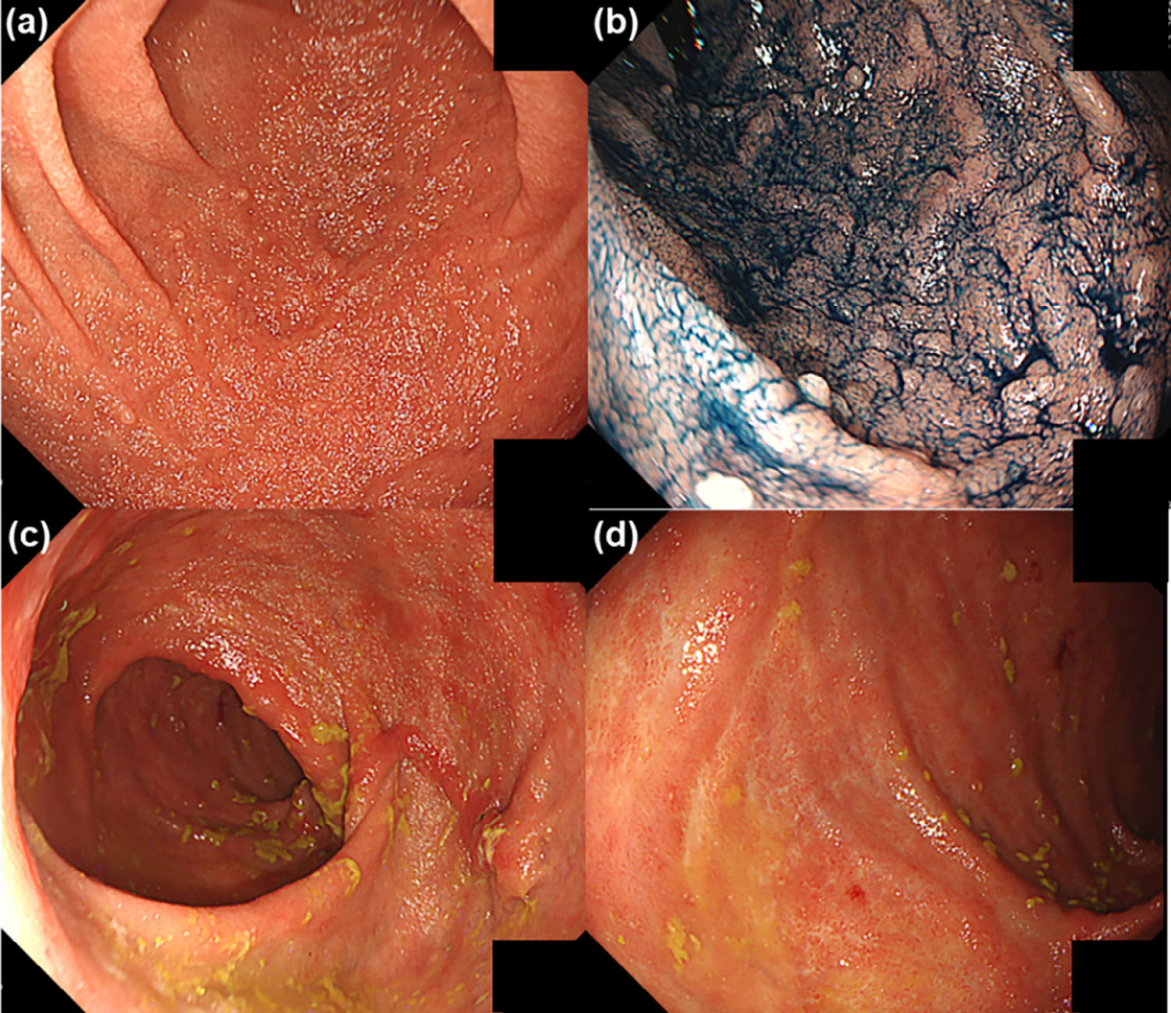
Esophagogastroduodenoscopy revealed scattered inflammatory polyps in the duodenum (a, b), and pouchcoscopy revealed that the ulcers and erosions had disappeared (c, d).

A 27‐year‐old man presented to our department with epigastric discomfort, increased stool frequency (>20 times a day), and bloody diarrhea for a fortnight. He had pancolitis‐type UC 1 year prior and underwent a colectomy and two‐stage ileal pouch‐anal anastomosis for medically refractory UC 6 months later. Esophagogastroduodenoscopy and pouchoscopy performed before stoma closure revealed no abnormal findings in the duodenum and focal inflammation of the pouch body (Figures 1a and 2a). Three months after the stoma closure, the gastrointestinal complaints occurred. Laboratory data revealed a C‐reactive protein (CRP) level of 6.9 mg/dL, an albumin level of 1.9 g/dL, a leucine‐rich alpha‐2 glycoprotein level of 54.8 µg/mL, and a hemoglobin level of 12.9 g/dL. Computed tomography revealed pouch edema. He was hospitalized due to oral intake difficulties. Esophagogastroduodenoscopy revealed continuous diffuse friable mucosa and edema in the duodenum (Figure 1b,c). Pouchoscopy revealed edema, friability, loss of vascular pattern, mucous exudates, and ulceration (the endoscopic subscore of pouchitis disease activity index of 5; Figure 2b,c). *Helicobacter pylori* immunoglobulin G antibody was negative. Stool cultures were negative for *Clostridioides difficile*. Cytomegalovirus was negative based on antigenemia assay and mucosal biopsy. Duodenum biopsy revealed diffuse inflammatory cell infiltration, cryptitis, and crypt abscesses, and pouch biopsy revealed numerous neutrophilic infiltrations (Figures 1d and 2d). Based on endoscopic and histopathological findings, the patient was diagnosed with duodenitis associated with UC and pouchitis (modified pouchitis disease activity index of 11). Treatment with oral prednisolone (40 mg/day) and ciprofloxacin was initiated (Figure 3). Two weeks after starting corticosteroid therapy, stool frequency and bloody stools decreased, and CRP levels normalized (0.05 mg/dL). However, when the prednisone dose was reduced to 10 mg/day, the frequency of bloody stools and the CRP level increased. When prednisolone was discontinued after 12 weeks of corticosteroid therapy, the symptoms worsened, albumin level decreased, and CRP level increased. We then administered 20 mg prednisolone sodium phosphate enema once daily and discontinued ciprofloxacin. One month later, symptoms and laboratory values improved. However, when the enema was discontinued, epigastric discomfort, bloody stools, and elevated CRP (0.75 mg/dL) recurred. We concluded that the patient had steroid‐dependent duodenitis associated with UC and pouchitis. UPA, a Janus kinase (JAK) inhibitor, was selected because preoperative treatments with infliximab, ustekinumab, vedolizumab, and tacrolimus were ineffective. Moreover, the patient preferred oral medications due to the difficulty in intravenous access. One month after starting UPA at 45 mg/day, epigastric discomfort and bloody diarrhea disappeared, and leucine‐rich alpha‐2 glycoprotein and CRP levels normalized (10.0 µg/mL and 0.04 mg/dL, respectively). A month later, the UPA dose was reduced to 30 mg/day, and one month after that, to 15 mg/day. No adverse events occurred, and the patient's symptoms remained in remission. Nine months after UPA initiation, esophagogastroduodenoscopy revealed scattered inflammatory polyps and no active inflammatory findings in the duodenum (Figure 4a,b). Pouchoscopy revealed remitted mucosa with vascular permeability, and the ulcers and erosions had disappeared (modified pouchitis disease activity index of 0; Figure 4c,d). The patient has been in clinical remission for 1 year without adverse events. We plan to continue UPA therapy.

## DISCUSSION

Herein, we report the case of a man who developed duodenitis associated with UC and pouchitis after a total colectomy. He developed severe corticosteroid‐dependent duodenitis and pouchitis. Clinical and endoscopic remissions without serious complications were achieved after UPA treatment. This is the first report on UPA efficacy in patients with upper UC lesions and pouchitis.

Local small intestine involvement, such as backwash ileitis and pouchitis, has been reported in patients with UC. However, UC‐related UGI is rare, and physicians do not recognize it. Diagnosis is difficult as no typical symptoms or histopathological features are present. Hori et al.^2^ called UC‐related UGI “gastroduodenitis associated with UC.” They reported that gastroduodenitis associated with UC prevalence was 7.6% and that pancolitis and a lower prednisolone dose were significant risk factors for developing gastroduodenitis associated with UC. Hisabe et al.^3^ called it “ulcerative gastroduodenal lesion” and reported a prevalence of 4.7%. In these reports, UC‐related UGI was only observed in patients with pancolitis or postproctocolectomy and was often complicated by pouchitis. Endoscopic findings include friable and granular mucosa and multiple aphthae. The first two were not observed in patients without UC; therefore, they were considered specific for UC‐related UGI.^2^ Additionally, it has recently been reported that preoperative severe disease activity may predispose postoperative duodenitis in UC patients.^6^ In this case, the preoperative partial Mayo score was 9, indicating high activity.

Currently, there are no established treatments for UC‐related UGI. Gastric secretion inhibitors such as histamine receptor antagonists and PPIs are ineffective, whereas 5‐aminosalicyclic acid and corticosteroids effectively treat UC.^3^ The efficacy of anti‐tumor necrosis factor‐α agents or calcineurin inhibitors has also been reported.^4^, ^5^ Although there are no reports on the efficacy of JAK inhibitors for UC‐related UGI, there have been several case reports for pouchitis. Akiyama et al.^7^ reported that the efficacy of tofacitinib for pouchitis was 46%, while Lan et al.^8^ reported that upadacitinib did not achieve sufficient efficacy. However, most of the patients with pouchitis in these studies had been exposed to biologics. The patient here had previously taken PPIs, but the symptoms were uncontrolled. Similar to colitis treatment, we initially used corticosteroids to induce remission as the patient had mesalamine intolerance and steroid‐dependent UC before a total colectomy. After the clinical symptoms and blood test results had improved, the symptoms relapsed when the corticosteroid dose was reduced. Due to the steroid‐dependent clinical course, we considered JAK inhibitors as the subsequent therapy because the patient had not responded to anti‐tumor necrosis factor‐α antibodies, an interleukin‐12 and interleukin‐23 antagonist, an α4β7 integrin inhibitor, and a calcineurin inhibitor preoperatively. Moreover, he had difficulty with intravenous access and preferred oral medication. A network meta‐analysis on the efficacy of advanced therapies for moderate to severe UC suggested that UPA might have the highest efficacy.^9^ Additionally, there are reports indicating rapid improvement in UC symptoms from the first day of administration.^10^ Since the patient was biologics/JAK‐naive for UC‐related UGI and pouchitis, and desired early symptom improvement, we chose UPA because it is considered to have high efficacy and rapid action among JAK treatments. We consider that UPA is an effective treatment; however, collecting data from a large number of cases is necessary.

In conclusion, this is the first report of duodenitis associated with UC and pouchitis after total colectomy successfully treated with UPA. When patients with UC complain of UGI symptoms, considering UC‐related UGI is essential. If UC‐related UGI is diagnosed, treatment should be initiated promptly, similar to that for colitis. Step‐up treatments, such as biologics and JAK inhibitors, should be started in cases refractory to conventional treatment.

## CONFLICT OF INTEREST STATEMENT

None.

## ETHICS STATEMENT

All procedures were performed in accordance with the ethical standards of the 1964 Declaration of Helsinki and its later amendments.
